# Manganese protects wheat from the mycotoxin zearalenone and its derivatives

**DOI:** 10.1038/s41598-019-50664-5

**Published:** 2019-10-02

**Authors:** Barbara Gzyl-Malcher, Elżbieta Rudolphi-Skórska, Apolonia Sieprawska, Maria Filek

**Affiliations:** 1 Faculty of Chemistry, Jagiellonian University, Gronostajowa 2, 30-387 Kraków, Poland; 2 Institute of Biology, Pedagogical University, Podchorążych 2, Kraków, 30-084 Kraków, Poland; 3 Polish Academy of Science, The Franciszek Górski Institute of Plant Physiology, Niezapominajek 21, 30-239 Kraków, Poland

**Keywords:** Biophysical chemistry, Membrane biophysics, Cell growth, Plant cell biology

## Abstract

Searching for factors that reduce zearalenone (ZEN) toxicity is an important challenge in wheat production, considering that this crop is a basic dietary ingredient. ZEN, absorbed by cells, is metabolized into α-zearalenol and α-zearalanol, and this study focused on the function of manganese ions as potential protectants against the mycotoxins. Stress effects were invoked by an application of 30 µM ZEN and its derivatives. Manganese ions were applied at 100 µM, not stress-inducing concentration. Importance of the biomembrane structures in the absorption of the mycotoxins was demonstrated in *in vitro* wheat calli and on model membranes. ZEN showed the greatest and α-zearalanol the smallest stressogenic effect manifested as a decrease in the calli growth. This was confirmed by variable increase in antioxidant enzyme activity. Mn ions added to the toxin mixture diminished stressogenic properties of the toxins. Variable decrease in total lipid content and the percentage of phospholipid fraction detected in calli cells exposed to ZEN and its metabolites indicated significance of the membrane structure. An analysis of physicochemical parameters of model membranes build from phosphatidylcholine, a basic lipid in native membranes, and its mixture with the tested toxins made by Langmuir technique and verified by Brewster angle microscopy, confirmed variable contribution of ZEN and its derivatives to the modification of membrane properties. The order of toxicity was as follows: ZEN ≥ α-zearalenol > α-zearalanol. Manganese ions present in the hydrophilic phase interacted with polar lipid groups and reduced the extent of membrane modification caused by the mycotoxins.

## Introduction

Infestation of crops by fungi, especially from the Fusarium species is currently a significant problem both in agronomic terms, due to reduced yields as well as consumption, as fungal metabolites accumulated in plants are then taken in with food by animals and humans. Especially such crops as wheat, maize, barley and oat are exposed to the toxic effects of *Fusarium*^1^. Observed symptoms of mycotoxins actions are mainly necrosis and chlorosis leading to wilts, and rot of stalk, root and leaf^2,3^. As was shown by numerous studies carried out in different geographic regions, fungal metabolites, especially zearalenone, can be accumulated in these cereals in quantities from about 2 to 3,000 μg/kg, including wheat, from about 2–250 μg/kg^4,5^. In plant tissues, the most frequently identified the ZEN-derivatives were zearalanone, α-, β-zearalenol, and α-, β-zearalanol^4,6^, however, also β-glucoside, β-glucopyranoside and sulfate metabolites were registered^7–9^. In wheat, mainly the α-ZEL was detected in the range 8–16 µg/kg, β-ZEL – 2–49 µg/kg^10^, α-ZOL – 12 µg/kg and β-ZOL – 14 µg/kg^11^ and ZEN-14Glc (17–104 µg/kg)^7^, whereas other ZEN-metabolites were analyzed in the relatively low amount.

The problem of mycotoxin accumulation in the tissues^12^, cells^13^ and cellular organelles^14,15^ of plants that constitute the basics of human diet has prompted the search for methods that prevent the absorption of these substances. Mycotoxins consumed with food accumulate in animal and human cells^16,17^, act as carcinogens and initiate pathogenic changes in various organs^18,19^. One of the concept describing the mechanism of toxic effect of zearalenone in plants is the initiating of oxidative stress, as the final stage of a series of reactions occurring in cells^20–22^. Oxidative stress stimulate the effects of which involve generation of reactive oxygen species, that damage cellular structures by oxidizing their important components (lipids, proteins, DNA^23^. The reactive oxygen species (ROS) mainly contain superoxide radicals (O_2_^−^), singlet oxygen (^1^O_2_), hydrogen peroxide (H_2_O_2_), and hydroxyl radical (OH^−^).

Natural protective mechanisms against excessive ROS presence include activation of enzymatic (SOD, CAT, POX) and non-enzymatic antioxidants. The different methods are used for detoxification of mycotoxins including the biological neutralization by microorganisms, mainly bacteria^24–28^, and saccharomyces^29^ as well as by physical^30,31^ and chemical^32^ adsorbents. In our previous works, it was demonstrated the reduced ZEN uptake resulting from both foliar administration and seed soaking in brassinosteroids (24-EBR) – phytohormones naturally occurring in cells, as well as from treatment with selenium ions, the accumulation of which is beneficial under oxidative stress conditions^14,15,33^. We suggested that brassinosteroids, with their hydrophilic-hydrophobic molecular structure similar to ZEN, may bind to the lipid part of the bilayer and displace ZEN from membranes. The protective role of selenium anions consisted in their interaction with water molecules and modulation of water molecular structure surrounding the polar moieties of lipids^34^. Such a modification of physicochemical properties of the membrane may reduce ZEN adsorption. The mentioned studies indicated the importance of the cell membrane composition and its structures as factors directly involved in the mechanism of ZEN intracellular transport.

Confirming protective properties of ions in the anionic form encouraged hypotheses on possible similar activity of cationic substances that may directly interact with negatively charged molecules (such as lipids) being the membrane components. This focused of our attention on manganese an element necessary for proper plant development. The Mn exhibits oxidation levels in the range Mn^−3^ to Mn^+7^ ^35^, however, in biological systems the predominant oxidation states are Mn^+2^ and Mn^+3^ ^36,37^. In aqueous solutions, Mn^+2^ is coordinated with HO^−^ or O^−2^ ions through oxygen atoms, and, in biological systems (mainly in chloroplasts and mitochondria), with amino acids (such as aspartate, glutamate, tyrosine) with coordination numbers 5 and 6, and less frequently 4^38^.

The studies of Mn action in cells has been concentrated rather to explain the physiological mechanism in which participate. The presence of these ions is especially important for the photosynthetic reactions and many enzymatic processes. In antioxidant enzymes (e.g. superoxide dismutase and catalase), Mn acts as a cofactor of protein enzymes^39,40^. Thus, Mn ions are indirectly involved in cell protection against oxidative stress. Mn cations may be directly uptaken from media via root system and translocated to all plant organs. Transmembrane protein carriers NRAMP2 specialize in Mn transport into cells^41^. The negative (summary) membrane charge promotes attraction of Mn cations, leading to electrokinetic modifications of the membrane surface. Such changes may affect adsorption of other substances in the vicinity of the membrane, including zearalenone.

**Figure 1:**
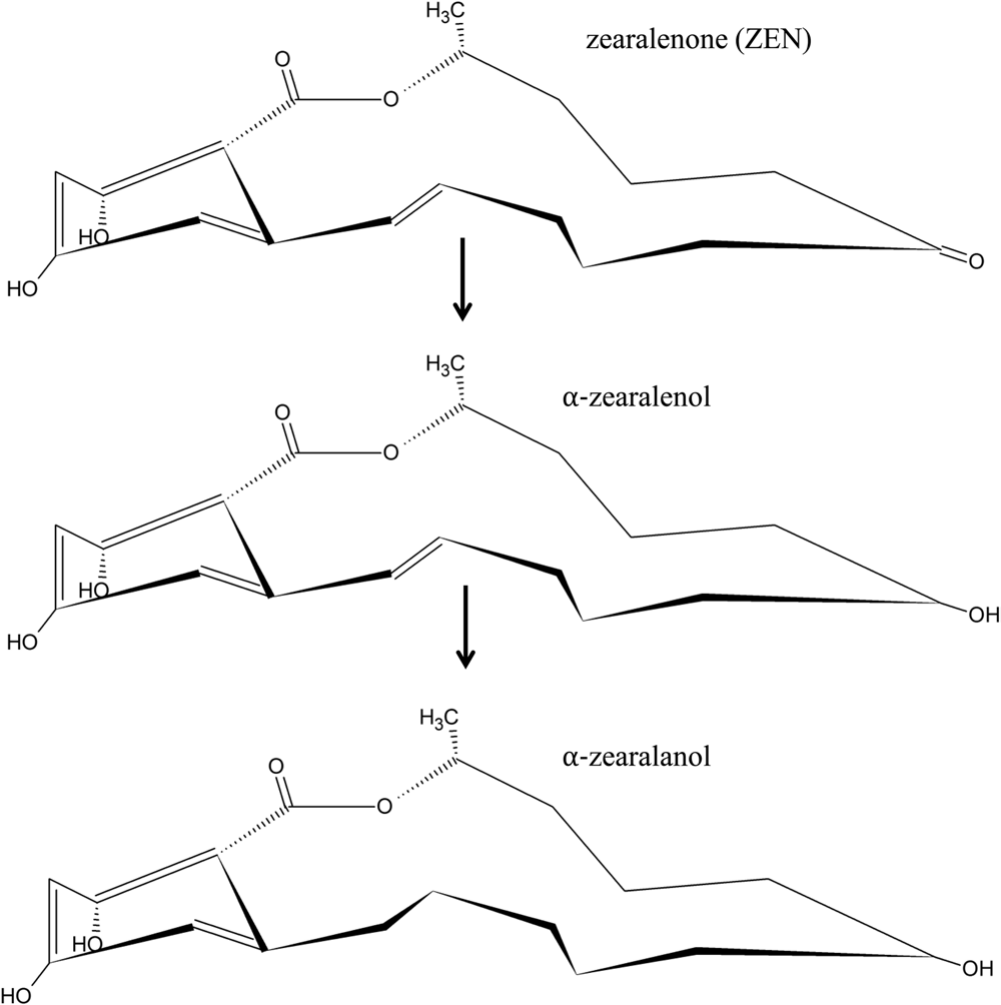
Chemical structure of zearalenone and its derivatives: α-zearalenol and α-zearalanol.

Zearalenone with its structure containing fourteen-membered lactone combined with 1,3-dihydroxybenzene reminds an estrogen molecule (Fig. 1), and displays a hormone-like effect in cells^42^. Gzyl-Malcher *et al*.^33^ demonstrated that hydroxyl, ketone or lactone groups of ZEN may form hydrogen bonds with polar lipid parts of membranes, and hydrocarbon rings modify the structure of their hydrophobic parts thereby changing the van der Waals interactions. After penetration into cells, ZEN undergoes biotransformation and yields derivatives (such as α-, β-zearalenol, α-, β-zearalanol) that differ in the arrangement of functional groups and double bonds (Fig. 1). This transformation can lead to changing in ZEN toxicity^43^.

The aims of these studies were (1) to verify whether the impact of ZEN derivatives may reduce the oxidative stress intensity in comparison with ZEN; and (2) to check the extent to which the addition of Mn ions may protect plants against ZEN and its derivatives. As Mn cations can interact directly with negative charged surface of the membrane this element offer better possibility of stabilizing the lipid structure under ZEN-evoked stress than the studied earlier Se ions (SeO_4_^2−^). The intensity of stress stimulated by the presence of tested mycotoxins was determined by physiological parameters (changes in growth mass) and biochemical (antioxidant enzyme activity). The effects of ZEN and its derivatives in the presence of Mn were verified directly in wheat calli cells cultured *in vitro* and by precise monitoring of physicochemical changes in model lipid membranes. Wheat calli cells, for which the stressogenic effect of ZEN was described in previous studies^15^, was selected to investigate the effects of mycotoxins on biochemical parameters of natural membranes.

To describe the mechanism of interaction of the tested substances with membranes, the experiments were performed also in model systems (Langmuir monolayers). Model studies of lipid monolayers, in which physicochemical parameters characterizing the membrane structure allow to determine subtle differences resulting from the lipid-tested substance interactions^44^. Visualization of monolayer microscopic structure by Brewster Angle Microscopy (BAM) allowed to demonstrates the formation of various lipid domains depending on the presence of substances added into the hydrophilic/hydrophobic phase.

## Results

### The influence of toxins alone and in the presence of Mn on the properties of native membranes

**Figure 2:**
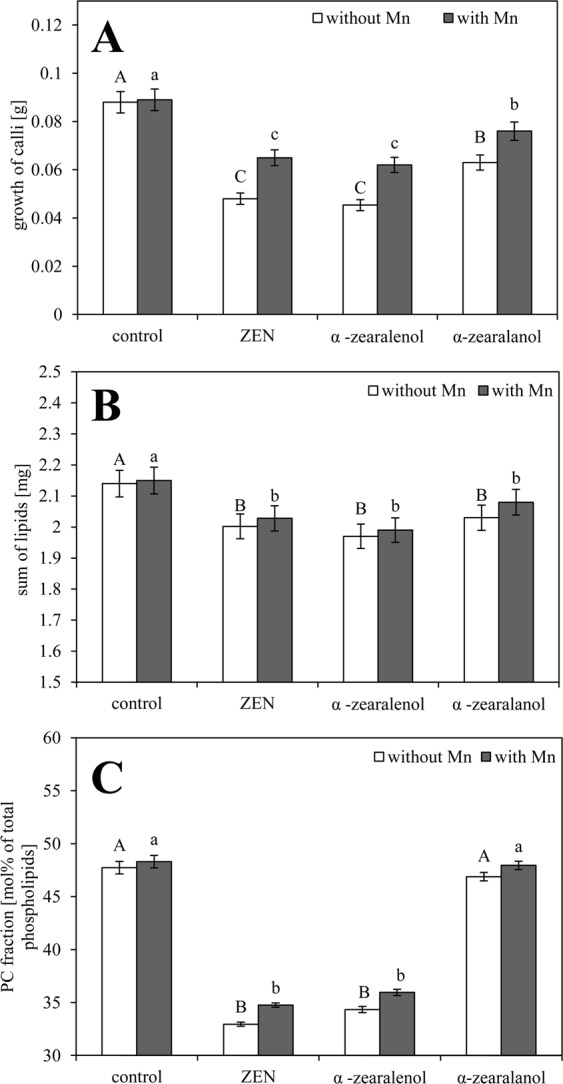
Growth of calli [g] (**A**), sum of lipids [mg/g of calli] (**B**), and content of PC fraction [mol % of total phospholipids] (**C**) in calli cells of Raweta wheat. Calli were cultured at MS media (control) and media with 30 µM ZEN, 30 µM α-zearalenol, 30 µM α-zearalanol without or with 100 µM Mn ions. Data are the means of ten replication with ± SE. Means followed by the same letters for each treatment are not significantly different (P < 0.05).

The introduction of ZEN and α-zearalenol to the culture media reduced the growth of cv. ‘Raweta’ calli by about 20–25%, depending on the toxin, and α-zearalanol induced the smallest changes in comparison with control (Fig. 2A). The presence of Mn did not change the cell growth parameters for the control media. For the media supplemented with Mn and ZEN or its derivatives it was noticed a significant increase in calli weight vs. the media with toxins alone.

Plasmalemma of toxin-treated calli contained less lipids (calculated to 1 g of fresh weight) than the control one. The drop reached about 7%, and Mn addition did not visibly change the lipid content (Fig. 2B). Phosphatidylcholine (PC) was the richest fraction in the phospholipid pool but during calli growth in the toxic environment the amount of this lipid class decreased by about 30% following ZEN and α-zearalenol application (Fig. 2C). The smallest, not significant modifications were observed for α-zearalanol treatment. Manganese addition increased PC levels only when the element was supplemented together with ZEN and α-zearalenol.

ZEN and α-zearalenol application enhanced the activity of all main antioxidant enzymes (SOD, POX, APX and CAT), as compared with control, whereas α-zearalanol did not significantly affect the enzymatic activity (Table 1). In general, Mn added together with the toxins slightly decreased the enzyme activity vs. the calli treated with toxins alone. In the control samples, Mn ions did not stimulate visible changes in the enzymes activity, which were similar to the control level.

**Table 1:** Superoxide dismutase (SOD), catalase (CAT), peroxidases (POX) and ascorbate peroxidase (APX) activity in calli cells of Raweta wheat of control and with ZEN, α-zearalenol, α-zearalanol at 30 µM and with using toxins in the mixture with 100 µM Mn.

	SOD [U/mg protein]	CAT [U/µg protein]	POX [U/mg protein]	APX [U/µg protein]
***Without Mn***				
Control	0.036 ± 0.004^c^	0.026 ± 0.003^b^	0.111 ± 0.008^b^	1.32 ± 0.02^c^
ZEA	0.065 ± 0.005^a^	0.038 ± 0.004^a^	0.137 ± 0.007^a^	1.54 ± 0.03^a^
α-ZEL	0.053 ± 0.003^b^	0.033 ± 0.002^a^	0.125 ± 0.007^a^	1.41 ± 0.02^b^
α-ZAL	0.041 ± 0.004^c^	0.029 ± 0.003^b^	0.117 ± 0.006^b^	1.35 ± 0.01^c^
***With Mn***				
Control	0.037 ± 0.003^c^	0.026 ± 0.002^b^	0.113 ± 0.004^b^	1.33 ± 0.02^c^
ZEA	0.052 ± 0.004^a^	0.035 ± 0.004^a^	0.129 ± 0.005^a^	1.48 ± 0.04^a^
α-ZEL	0.045 ± 0.002^b^	0.030 ± 0.002^a^	0.120 ± 0.004^a^	1.39 ± 0.02^b^
α-ZAL	0.038 ± 0.003^c^	0.027 ± 0.003^b^	0.112 ± 0.003^b^	1.34 ± 0.02^c^

Data are the means of ten replication with ± SE. Means followed by the same letters for each treatment are not significantly different (p < 0.05).

### The influence of toxins and Mn on the model membranes properties

#### Isotherms of surface tension (π) versus molecule areas (A)

**Figure 3:**
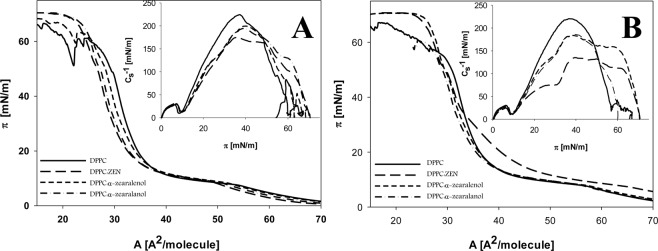
Isotherms of surface tension (π) versus area per molecule (**A**) for monolayers prepared with DPPC and DPPC + toxin mixture (at a molar ratio of 4:1) on the surface of pure water (**A**), and surface of 100 µM of MnCl_2_ solution. (**B**) Insert – values of C_s_^−1^ versus surface pressure (π) are introduced.

Isotherms of model monolayers of phosphatidylcholine (DPPC) and its mixture with studied toxins on the surface of pure water and surface of Mn solution are presented on the Fig. 3A,B, respectively. Isotherms of pure DPPC monolayers shown the course characteristic for this class of lipids with plateau indicating the phase transitions. The presence of the studied toxins in the mixture with DPPC exerted only small effects on the shape of this plateau when in contact with the water subphase (Fig. 3A). In the water solution supplemented with Mn, the plateau of DPPC + ZEN almost disappeared, while for the other studied systems the Mn triggered changes were smaller and remained close to those detected for DPPC alone. The values of structural parameters calculated from isotherm data were shown in Table 2.

**Table 2:** The surface area per single lipid molecule of the maximum packed layer A_lim_ [Å^2^], π_coll_ [mN/m] parameter and the maximum compression module C_s_^−1^_max_ [mN/m] obtained for monolayers prepared with DPPC and mixtures of DPPC with the studied toxins in phase contact with water and Mn^2+^ water solution.

System	A_lim_ [Å^2^]	π[mN/m]	C_s_^−1^_max_ [mN/m]
**Pure water as a subphase**			
DPPC	36.11 ± 0.04^d^	43.85 ± 0.05^a^	223.77 ± 0.06^a^
DPPC + ZEN	39.42 ± 0.03^a^	36.53 ± 0.03^d^	174.58 ± 0.07^d^
DPPC + α-zearalenol	38.60 ± 0.02^b^	38.94 ± 0.04^c^	194.34 ± 0.05^c^
DPPC + α-zearalanol	37.63 ± 0.04^c^	39.57 ± 0.03^b^	198.79 ± 0.08^b^
**100** **µM MnCl**_**2**_ **solution as a subphase**			
DPPC	37.62 ± 0.02^c^	36.60 ± 0.03^c^	219.21 ± 0.07^a^
DPPC + ZEN	38.71 ± 0.04^a^	40.31 ± 0.04^a^	134.66 ± 0.08^d^
DPPC + α-zearalenol	38.10 ± 0.03^b^	39.90 ± 0.03^a^	186.85 ± 0.09^b^
DPPC + α-zearalanol	37.55 ± 0.05^c^	39.58 ± 0.02^b^	184.76 ± 0.06^c^

Data are the means of three replications ± SE. Means followed by the same letters for each parameter are not significantly different (P < 0.05).

The toxin presence in the DPPC monolayer increased the surface area per single lipid molecule in the maximum packed layer (A_lim_) by about 8%, 7% and 4% for ZEN, α-zearalenol and α-zearalanol, respectively. Introducing of Mn ions resulted in the increase of A_lim_ values for DPPC and decrease for DPPC + toxins mixtures vs. data for pure water, with the greatest changes for ZEN (about 2.5 Å^2^). For DPPC monolayers containing the remaining toxins, the modifications reached about 1.4–2 Å^2^.

At the point where the tested monolayers were compressed to the most compacted monolayers, the isotherms of DPPC + toxins broke down at lower surface tension than DPPC alone, which was precisely indicated by π_coll_ parameter. The changes were the most pronounced (relative to DPPC) for ZEN in the lipid mixture and least visible for α-zearalanol. For the Mn solution subphase, the π_coll_ values of DPPC monolayers decreased (by about 16%) in comparison with those spread on pure water. When monolayers were prepared from DPPC + ZEN mixtures, π_coll_ parameter increased by about 10% vs. the layer on water. For the other studied mixtures, the presence of Mn ions did not evoke clear changes of this parameter.

Calculation of maximum compression module (C_s_^−1^_max_) parameters based on Fig. 3A,B demonstrated the highest values of this factor for pure DPPC (Table 2). Toxins present in DPPC monolayers significantly reduced this parameter, with the greatest drop for ZEN. The changes were visible both on water and Mn containing solution but the addition of manganese ions into the subphase significantly enhanced this effect.

#### Gibbs free energy

The isotherms allowed to calculate the Gibbs free energy changes of DPPC monolayers modified by zearalenone or its metabolites presence, according to the formula of Dei *et al*.^45^:

$$\Delta G={\int }_{0}^{{\pi }_{0}}\,{A}_{1}\,d\pi -{\int }_{0}^{{\pi }_{0}}\,{A}_{0}\,d\pi $$

The surface pressure π_0_ = 20 [mN/m] is the upper limit pressure used to calculate the integral, A_1_ is experimental area as function of surface pressure of DPPC in presence of analysed toxins, A_0_ is experimental area as function of surface pressure for DPPC.

The resulting values were used to quantify the effect of the tested toxins on the thermodynamic stability of monolayers prepared on the water and Mn solutions phases (Table 3). The changes of Gibbs energy for the studied systems of phospholipid and phospholipid + toxin monolayers were compared to describe the thermodynamic effects connected with: (1) the presence of various derivatives of zearalenone on the condition of DPPC layer in contact with pure water and (2) with the subphase containing manganese solution. I was also evaluated (3) how Mn supplementation modified the Gibbs energy of monolayers vs. the effects observed on pure water.

**Table 3:** Gibbs free energy values calculated for monolayers of DPPC + toxins remaining with the phase contact with pure water and with MnCl_2_ (100 µM) solution.

System	ΔG [kJ/mol]	
DPPC monolayers+	subphase	
*toxins*	water	Mn
ZEN	−0.35 ± 0.02^a^	−3.30 ± 0.03^c^
α-zearalenol	−0.39 ± 0.02^b^	−1.44 ± 0.02^b^
α-zearalanol	−0.74 ± 0.01^c^	−1.21 ± 0.01^a^

Data are means of three replications ± SE. Means followed by the same letters for each subphase are not significantly different (P < 0.05).

The presence of ZEN and its derivatives in DPPC monolayer remaining in contact with both water and Mn phase decreased the Gibbs energy, related to DPPC alone, expressed as negative ΔG values (Table 3). However, when pure water interacted with monolayers, the strongest effect (more negative ΔG values) was observed for α-zearalanol. In Mn solutions ZEN application caused the greater decrease of ΔG values. Moreover, the changes of ΔG values were more visible when Mn ions were dissolved in water. Analysis of the effects of these ions in relation to pure water confirmed that the biggest modification resulting from different composition (Mn addition) of the hydrophilic layer was caused by zearalenone + DPPC combination (Table 4). The values of ΔG for the other tested mixtures were closer to those calculated for DPPC alone.

**Table 4:** The influence of Mn presence vs. pure water on the thermodynamic condition of interphase (indicated as changes of free Gibbs energy).

DPPC monolayers	ΔG [kJ/mol]
toxins	
0	−1.16 ± 0.02^a^
ZEN	−4.12 ± 0.05^d^
α-zearalenol	−2.21 ± 0.03^c^
α-zearalanol	−1.63 ± 0.02^b^

Data are means of three replications ± SE. Means followed by the same letters for each subphase are not significantly different (P < 0.05).

#### BAM observations

**Figure 4:**
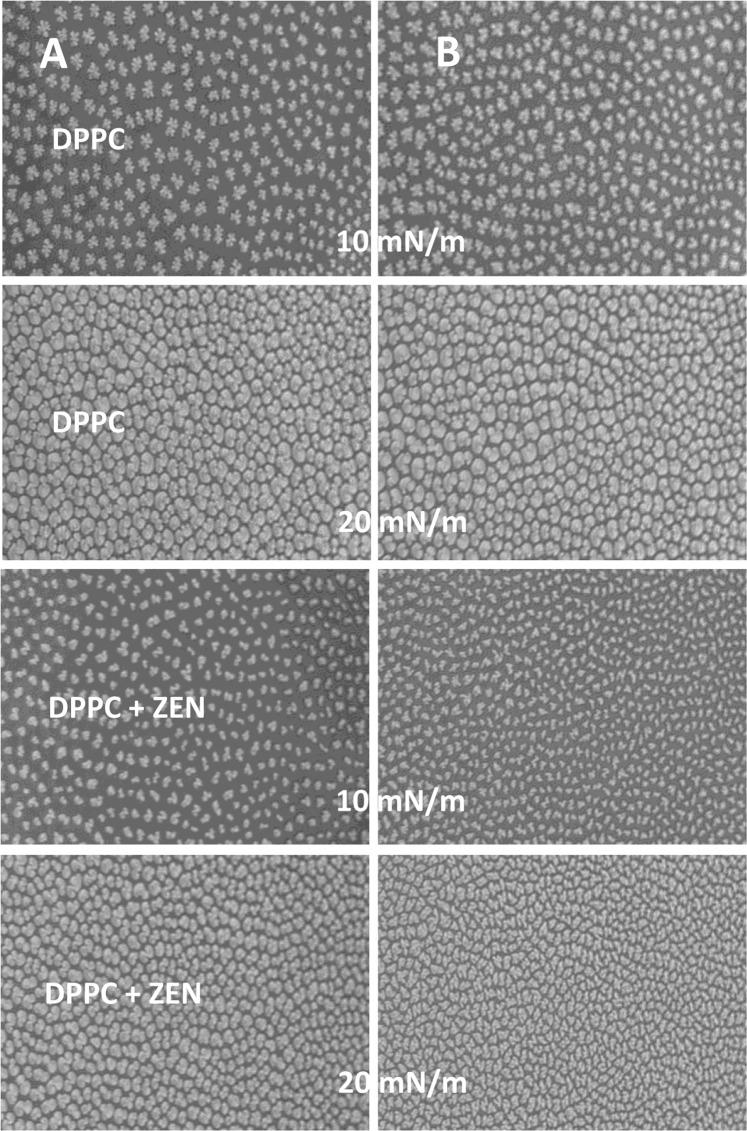
Domain patterns formed in DPPC and DPPC + zearalenone (DPPC + ZEN) monolayers for surface pressures of 10 and 20 mN/m in absence (**A**) and in presence of 100 µM Mn (**B**).

**Figure 5:**
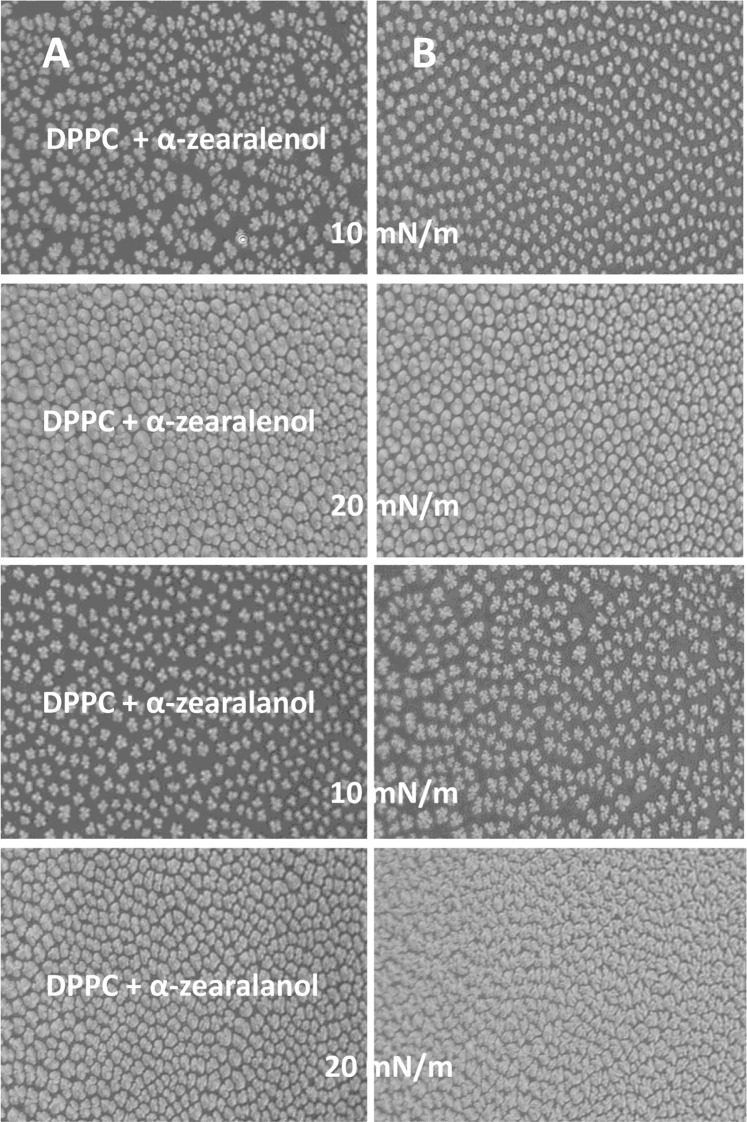
Domain patterns formed in DPPC + α-zearalenol and DPPC + α-zearalanol monolayers for surface pressures of 10 and 20 mN/m in absence (**A**) and in presence of 100 µM manganese (**B**).

Morphology of the monolayers was recorded by the BAM method at π = 10 mN/m and 20 mN/m – the values that corresponded to gradual packing of molecules during monolayer compression (Figs 4 and 5). During compression of DPPC layer light gray structures (organized in multi-lobed-domains) of varying numbers, shapes and sizes appeared at the water surface. The occurrence of these structures corresponds with the phase transition during monolayer shift from liquid-expand to liquid-condensed. The morphology of the created domains depended on the composition of the monolayer and/or the composition of the subphase. The DPPC domains obtained on water surface (Fig. 4A) at 10 mN/m had a characteristic multi-lobed shape, and they were uniform in size. Further compressing increased both the size and shape of the domains. Introduction of Mn^2+^ (Fig. 4B) into the water phase did not affect the morphology of the DPPC domains but they become less organized and had smoother contours. Domains formed in the presence of ZEN mixed with DPPC were of much smaller size and less regular shape. There were bean- and two-lobed-shaped-domains, which at a higher pressure (20 mN/m) were organized into more homogeneous, slightly larger aggregates with smoother contours. Nevertheless, they were much smaller when compared with DPPC alone. The same monolayer (DPPC + ZEN), but formed on Mn^2+^ subphase, had a completely different organization. The domains were smaller, more irregular, and with sharp contours.

In the DPPC + α-zearalenol system (Fig. 5A), the emerging domains also displayed a multi-lobed organization but their lobes were less regular. Moreover, they differed in size and during compression the area of aggregates increased showing the rounded borders. The presence of Mn in the subphase did not affect the shape of the domains but they become more uniform in size (Fig. 5B). For α-zearalanol + DPPC combination, the domain morphology was very similar to that for DPPC alone but smaller objects also appeared. Manganese ions significantly changed the shape of the domains that also formed more complex structures (multi-lobed). During compression to 20 mN/m, these structures still showed sharp contours.

## Discussion

Studies on the role of mycotoxins in cells are run in various aspects among other to describe the mechanisms involved in their metabolism (after penetration into cells), to reduce their toxicity, such as binding to beta-D-Glucan^46,47^, as well as finding methods of preventing (reducing) their uptake by cells. The presented experiments focused on demonstrating the significance of the chemical structure of ZEN and its main derivatives in modifying interactions with cellular membranes to find the differences in the possibility of membrane penetration by these compounds and to check the eventual protective effect of exogenous use of Mn ions against these mycotoxins. ZENs located in the lipid part of the membrane may (like sterols) cause a formation of specific domains, which affect the mechanical properties (stiffness, semi-liquidity) of membranes, which in turn may also modify the structure of transmembrane proteins (receptors, transporters and enzymes), stimulate/inhibit their activity. The possibility of impact of ZEN on the changes of lipid structure of native membranes has been suggested in earlier studies^33^.

Decrease of the calli weight in the presence of ZEN and its derivatives confirmed toxicity of the investigated substances, as the changes in growth parameters serve as indicators of plant stress^48,49^. The selected 30 µmol.dm^−3^ concentration was a threshold value at which ZEN was stressogenic for cells but did not significantly affect membrane integrity^20,50^. α-Zearalenol, an early metabolite of ZEN degradation in cells, exerted stress effects of similar strength to ZEN, whereas α-zearalanol, one of the final products of ZEN decomposition, was the weakest stressor. Analyzes of the activity of antioxidant enzymes in callus cultures, which were grown in the presence of Mn ions, indicate that the applied Mn ion concentration did not affect the metabolism in wheat cells. In the mixture with tested mycotoxins, Mn ions stimulated a partial reduction of ZEN and its derivatives toxicity. The lowered activity of SOD, POX, APX and CAT in the presence of Mn ions confirmed its role in defense mechanisms, especially against ZEN and α-zearalenol induced stress, as the decrease in antioxidant activity results from diminished ROS generation. We focused on the activity of this group of enzymes, although Mn is also mediating the activation of other classes of enzymes, mainly that found in chloroplasts^38^. A smaller direct participation of Mn in the activation of SOD (comparison of the results for cells cultured on controls media without and with Mn), in which one of the form is MnSOD, as well as the lack of chloroplasts in undifferentiated callus cultures prompted us to the consideration of the role of Mn ions in activation of antioxidant enzymes.

In our additional studies on the influence of high doses of Mn on the activity of SOD (data in preparation) the increase of SOD activation (also Mn-SOD form) was observed. The lack of differences in SOD activities, shown in the present study, for control cells cultured on the media without/with Mn, may indicate that the given Mn concentration does not stimulate ROS generation (does not initiate oxidative stress). Diminished of antioxidative enzymes activity (including SOD) in toxin + Mn test systems, compared to systems in which only mycotoxins were present, indicates a decreasing of ROS production, and reduction of oxidative stress conditions. We suggest that the protective role of Mn against the tested toxins may be a result of Mn-initiated modifications of the structural and physicochemical parameters of membranes limiting the penetration of toxins into the cells.

The decrease of lipid level in plasmalemma and amounts of PC fraction in studied calli cells registered in the presence of ZEN and its derivatives indicate the influence of the toxins on chemical properties of the membrane. ZEN-stimulated reduction of phospholipids (PL) pool in wheat calli was also registered in our earlier studies^33^. Maejima and Watanabe^51^ postulated that the decrease of PL concentrations under stress serves as one of the protective mechanisms activated in plant cells. The correlation between the stressogenic action of the toxins manifested as changes in PC content may point out to the validity of the membrane composition in the interactions with mycotoxins.

The precise description of the changes in membrane properties modified by tested substances was performed in experiments of model lipid monolayer systems. Our earlier work showed that ZEN molecule was able to penetrate the phospholipid monolayers and that this ability depended on the fatty acids saturation responsible for hydrophobic properties of the lipids^33^. In presented experiments, DPPC - that contains saturated fatty acids (16:0), was used to create a model of a lipid membrane. Such a layer provided more reliable observations of subtle changes in physicochemical properties modulated by incorporation of amphiphilic molecules. Van der Waals interactions between saturated fatty acids determine rigidity of the hydrophobic part of the layer, and incorporation of an additional molecule may cause a larger disturbances in the monolayer structure than in the presence of unsaturated acids (where the monolayer is more loosely packed).

**Figure 6:**
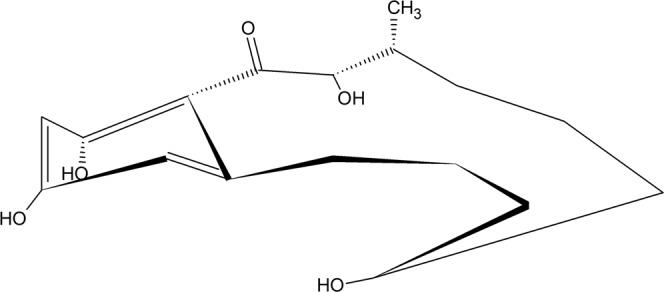
The suggested structure of the α-zearalenone molecule.

The calculation of structural parameters of DPPC monolayer (**A**_**lim**_, **π**_**coll**_, **C**_**s**_^**−1**^**)** allowed us to rank toxicity of the investigated substances in the following order: ZEN ≥ α-zearalenol > α-zearalanol. The strongest toxicity of ZEN manifested as a meaningful increase in the distance of lipid molecules in the DPPC monolayer (A_lim_), and related reduction in the monolayer stiffness and tightness (π_coll_, C_s_^−1^), as well as the weakest toxicity determined for α-zearalanol, correlated with the toxin effects observed in the calli cells. The expansion of the model monolayers induced by ZEN was also reported by Gzyl-Malcher *et al*.^33^ and Filek *et al*.^15^. Different interactions of the studied toxins with DPPC probably result from differences in their structure. ZEN and α-zearalenol molecules have a similar chemical structure with a double bond between carbon 1′ and 2′. A substitution of an oxygen atom by OH group at carbon 6′ in α-zearalenol may enhance its reactivity (e.g. hydrogen bond formation) to a greater degree than in ZEN. This may result in transition of α-zearalenol molecules from the hydrophobic part of the monolayer towards its polar part. The single bond (between C 1′ and 2′) in α-zearalanol molecule makes it more flexible (Fig. 6) and may reduce its actual size during placement in the hydrophobic part of DPPC monolayer. Such hypotheses may be suggested by a reduction of the distance between lipid molecules (A_lim_) in α-zearalanol presence vs. to the values characteristic of DPPC with other studied toxins.

Differences in the interactions of the toxins + DPPC and the impact of these interactions on the organization of the layer was confirmed by observing changes in the shape of domains using the BAM technique. A comparison of changes in the domain shape confirmed our earlier conclusion of the strongest influence of ZEN on the intermolecular interactions in membranes.

The presence of Mn ions resulted the “compressing” of toxins + DPPC layers, as evidenced by reduced A_lim_ and lowered C_s_^−1^_max_ and π_coll_, vs. data for monolayers formed on pure water. An increase in A_lim_ value of DPPC alone in the presence of Mn suggests that these ions, interacting with the polar group of the lipid, may be partly incorporated into the monolayer structure. Differences in the shape of domains formed in the presence of Mn in comparison with those formed in water-based monolayers confirmed the interaction of Mn with lipids. The strongest effects of ZEN and the weakest of α-zearalanol manifested the role of the toxin structure in changing physiochemical properties of the monolayers.

The effects of the mycotoxins on the stability of DPPC monolayers were also evaluated by calculating the Gibbs energy expressed as a difference between the changes in the strange of interactions between the mixed layers and DPPC alone. Negative values of ΔG indicate strong interactions between monolayer-forming molecules^52^. We found the most intensive interactions for α-zearalanol and the weakest for ZEN presence in lipids where DPPC was in contact with pure water phase. This confirmed that ZEN destabilized monolayers structure to the greatest extent among the investigated substances. Mn addition changed these interactions and ZEN action vs. other toxins. The differences in the Gibbs energy resulted probable from strong influence of Mn ions on the polar lipid part, and modulation the structural properties of membranes to reducing the toxic effects of mycotoxins action. Manganese ions interacting strongly with polar lipid groups create a greater barrier to the penetration of zearalenone and its derivatives into membranes than pure water.

## Conclusions

The study demonstrated growth reduction of wheat calli treated with both ZEN and its metabolites. The mycotoxins triggered oxidative stress as shown by the activation of antioxidant enzymes, and based on these reactions the order of their toxicity was established as: ZEN ≥ α-zearalenol > α-zearalanol. The role of the mycotoxin chemical structure in their interactions with lipid membranes was determined in model studies. The presence of more flexible single bonds in α-zearalanol molecule instead of double bonds typical of ZEN and α-zearalenol was probably the reason of the weakest interference of this metabolite with the lipid structure in contact with water phase. Mn ions stabilized the polar part of membranes and modified their interactions with mycotoxins, thus acting as potential protectors especially against ZEN and α-zearalenol.

## Materials and Method

### Chemicals

Zearalenone, α-Zearalenol, α-Zearalanol and manganese chloride were purchased from Sigma-Aldrich Company (Germany, Munich). 1,2-dipalmitoyl-sn-glycero-3-phosphocholine (DPPC) were obtained from Avanti Polar Lipids (USA, Alabaster). Chloroform (Merc, Germany, Munich) was the spreading solvent. Water, which was used as a subphase in the model studies was re-distilled and purified by a Milli-Q system, with a specific resistance above 18.2 MQ cm^−1^.

All other reagents used for biochemical analysis were obtained from Sigma-Aldrich Company (Germany, Munich).

### Plant material

Seeds of the spring wheat cv. ‘Raweta’, were obtained from the Polish Plant Breeding Stations-Strzelce (Poland). This cultivar was indicated as a sensitive into oxidative stress in our earlier studies^53^. The seeds after sterilization with 80% ethanol and 10% perhydrol were germinated in dark (2 days) and next planted into pots with a mixture of soil:peat:sand (3:2:1; v/v/v). Seedlings were cultivated in a greenhouse at temperature (20/17 °C; day/night), and light [16/8 h day/night photoperiod, 1000 µmol (photon) m^−2^ s^−1^ light] until the first anthers revealed. Immature embryos, after isolation, were put into *in vitro* conditions. As the growth media the Murashige and Skoog nutrients^54^ with 2 mg/cm^3^ 2.4-D (2.4- dichlorophenoxyacetic acid) (MS) were used. Non-embryogenic calli (after about 3 months growth) in amounts of 1 g/Petri-dishes were transferred to MS media with 30 µM zearalenone (ZEN), 30 µM α-zearalenol, 30 µM α-zearalanol and media contains of these toxins with addition of 100 µM MnCl_2_. These concentrations of zearalenone were chosen as stressful for cells^20,50^, while Mn concentrations did not cause toxic effects^55^. After 10 days, the fresh weight of calli, from each dish, was detected. For biochemical analysis, samples of calli were frozen and stored at −80 °C.

### Antioxidant enzyme extraction and assays

1 g of calli were homogenized in 1.5 cm^3^ of 0.1 M potassium phosphate buffer at pH 7.8 containing 2 M a-dithiothreitol, 0.1 M EDTA, 1.25 M polyethylene glycol and ethylenediaminetetraacetic acid. The samples were centrifuged at 14 000 g for 10 min at 4 °C and the supernatant purified on a PD10 column (Amersham Biosciences, Sweden). The supernatant was applied to determine the enzyme activity and protein content. Protein concentration was analyzed according the procedure of the Bradford method^56^. Superoxide dismutase (SOD, EC 1.15.11) activity was analyzed spectrophotometrically at λ = 595 nm using the protocol described by McCord and Fridovich^57^. The activity of peroxidases (POX) was measured at λ = 485 nm by modified method of Luck^58^ and ascorbate peroxidase (APX) at λ = 290 nm^59^. Catalase (CAT) activities was detected at λ = 240 nm in accordance with the procedure described by Aebi^60^.

### Lipid extraction from the plasmalemma of calli

Plasmalemma was isolated according to the method described in detail by Gzyl-Malcher *et al*.^33^ and next the total content of membrane lipids was extracted^61^. Phospholipid fractions were separated from the other lipids using adsorptive and distributive column chromatography on silica acid. The qualitative and quantitative composition of this lipid class was detected by thin-layer chromatography^62^.

### Model membranes

#### Langmuir monolayers

As the phosphatidylcholine was the most abundant phospholipid fraction in studied calli, to the model studies DPPC with precisely defined both polar and hydrophobic structure, was chosen. The experiments were performed using the Langmuir technique (Minitrough, KSV, Finland). The monolayers were prepared by spreading of the chloroform solutions of DPPC or DPPC + toxin mixtures (at a molar ratio of 4:1) on the surface of water or MnCl_2_ (100 µM) aqueous solutions. The monolayers were compressed at a rate of 3.5–4.6 Å^2^/molecule × min. The experiments were repeated three or four times to ensure a high reproducibility of the obtained isotherms to ±0.1–0.3 Å^2^. The dependence of surface pressure (π) versus the area per lipid molecule (A) was the basis for obtaining the parameters characterizing the lipid monolayer structure such as: A_lim_ – the minimum area occupied by a single molecule in a fully packed layer, π_coll_ – pressure at which a layer collapses and C_s_^−1^ – static compression modulus representing the mechanical resistance against the layer compression that provides information on the stability and fluidity of a layer. Surface pressure was detected with accuracy of ±0.1 mN/m using a Platinum Wilhelmy plate connected to an electrobalance. All of the experiments were performed at 25 °C.

#### Brewser angle microscopy (BAM)

The morphology of monolayers was visualized using the Brewster angle microscope (ultraBAM, Accurion GmbH, Goettingen, Germany) equipped with a 50 mW Laser-emitting p-polarized light of 658 nm wavelength directed to the air/water interface at the Brewster angle (53.2°), 10x magnification objective, analyzer, polarizer and camera. The resolution of the obtained image was 2 µm. The BAM apparatus was installed to KSV 2000 Langmuir trough of total area 700 cm^2^ (Helsinki, Finland) set on the antivibration table with an active vibration isolation system.
